# Clinical Outcomes of Drug-Eluting versus Bare-Metal In-Stent Restenosis after the Treatment of Drug-Eluting Stent or Drug-Eluting Balloon: A Systematic Review and Meta-Analysis

**DOI:** 10.1155/2020/8179849

**Published:** 2020-06-26

**Authors:** Yi-Xing Yang, Yin Liu, Chang-Ping Li, Peng-Ju Lu, Jiao Wang, Jing Gao

**Affiliations:** ^1^Tianjin Medical University, No. 22 Qi Xiang Tai Road, Heping District, Tianjin 300070, China; ^2^Department of Cardiology, Tianjin Chest Hospital, No. 261 Tai Er Zhuang Road, Jinnan District, Tianjin 300222, China; ^3^Cardiovascular Institute, Tianjin Chest Hospital, No. 261 Tai Er Zhuang Road, Jinnan District, Tianjin 300222, China

## Abstract

**Background:**

Although drug-eluting stents (DES) have reduced the rates of in-stent restenosis (ISR) compared with bare-metal stents (BMS), DES related ISR (DES-ISR) still occurs and outcomes of DES-ISR remain unclear. The objective of this meta-analysis was to investigate the long-term clinical outcomes of patients with DES-ISR compared with patients with BMS related ISR (BMS-ISR) after the treatment of DES or drug-eluting balloon (DEB). *Methods and results*. We searched the literature in the main electronic databases including PUBMED, EMBASE, Cochrane Library, and Web of Science. The primary endpoints were target lesion revascularization (TLR) and target vessel revascularization (TVR). The secondary endpoints included all cause death (ACD), cardiac death (CD), myocardial infarction (MI), stent thrombosis or re-in-stent restenosis (ST/RE-ISR), and major adverse cardiovascular events (MACEs). A total of 19 studies with 6256 participants were finally included in this meta-analysis. Results showed that the rates of TLR (*P* < 0.00001), TVR (*P* < 0.00001), CD (*P*=0.02), ST/RE-ISR (*P* < 0.00001), and MACEs (*P* < 0.00001) were significantly higher in the DES-ISR group than in the BMS-ISR group. No significant differences were found between the two groups in the rates of MI (*P*=0.05) and ACD (*P*=0.21).

**Conclusions:**

Our study demonstrated that patients with DES-ISR had worse clinical outcomes at the long-term follow-up than patients with BMS-ISR after the treatment of DES or DEB, suggesting that DES and DEB may be more effective for BMS-ISR than that for DES-ISR. Positive prevention of DES-ISR is indispensable and further studies concentrating on detecting the predictors of outcomes of DES-ISR are required.

## 1. Introduction

Although the use of drug-eluting stents (DES) has significantly reduced the rates of in-stent restenosis (ISR) compared with bare-metal stents (BMS) [1,2], DES related ISR (DES-ISR) still occurs and the prognosis of patients with DES-ISR, which may be different from patients with BMS-ISR due to the different pathological features, remains unclear [3,4]. Recently, several studies investigated the long-term clinical outcomes of DES-ISR versus BMS-ISR after treated by DES or drug-eluting balloon (DEB), but the results were inconsistent [7–25]. Therefore, we enrolled these studies to conduct a meta-analysis to evaluate the results.

## 2. Methods

We searched the relevant literature in the main electronic databases (PUBMED, EMBASE, Cochrane Library, and Web of Science), using combinations of the following key words: “outcome” OR “prognosis” OR “result” AND “in-stent restenosis” OR “bare-metal in-stent restenosis” OR “drug-eluting in-stent restenosis.” Two authors independently performed the studies selection according to the titles or abstracts first, and then full texts of the relevant articles were evaluated according to the selection criteria. The inclusion criteria were as follows: (1) studies comparing the clinical outcomes of DES-ISR versus BMS-ISR; (2) treatment for ISR being DES or DEB; (3) follow-up time of at least six months; (4) studies including at least 30 participants; and (5) randomized clinical trials or observational studies. The exclusion criteria were as follows: (1) studies not comparing the clinical outcomes of DES-ISR versus BMS-ISR; (2) treatment for ISR including bare-metal stent (BMS), balloon angioplasty (BA) or coronary artery bypass surgery; (3) follow-up time being less than six months; (4) participants less than 30; and (5) case reports, reviews, and comments. A study was enrolled for the meta-analysis if it was eligible. Furthermore, we also searched the reference lists of all identified literatures to retrieve additional articles.

Two investigators independently performed the data extraction, using a standardized data extraction form including the following information: first author, year of study, type of study, number of participants, treatment of ISR, follow-up time, outcomes of ISR, and baseline characteristics of the enrolled patients. We tried to contact the authors by e-mails for the required data which was missing from the original published articles. Two reviewers independently assessed the risk of bias by using the Newcastle-Ottawa Scale [5]. Discrepancies were resolved by team discussion.

The primary endpoints were target lesion revascularization (TLR) and target vessel revascularization (TVR). The secondary endpoints included all cause death (ACD), cardiac death (CD), myocardial infarction (MI), stent thrombosis or re-in-stent restenosis (ST/RE-ISR), and major adverse cardiovascular events (MACEs). ISR is defined as recurrent diameter stenosis >50% at the stent segment or its edges. ACD is defined as death due to any cause. The definitions of “TLR,” “TVR,” “CD,” “MI,” “ST,” and “MACEs” were in accordance with the Academic Research Consortium criteria [6].

Effect sizes expressed as risk ratios (RR) with 95% confidence intervals (CIs) were calculated for each study. Statistical heterogeneity was evaluated by the Cochrane *Q* test and the *I*^2^ statistic. A random effect model was utilized if *P* values <0.1 and *I*^2^ values > 50%; conversely, a fixed effect model was used. Furthermore, to investigate the potential heterogeneity across studies, we also conducted subgroup analyses based on the treatment of ISR (DES or DEB). All analyses were carried out by the REVIEW MANAGER VERSION 5.3.

## 3. Results

**Figure 1:**
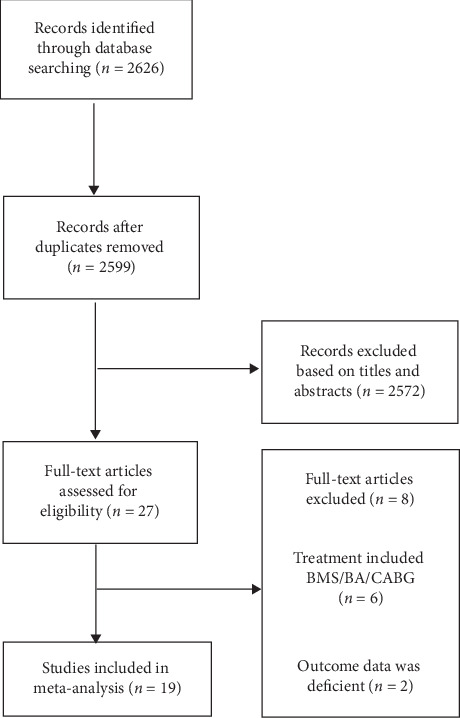
Flow diagram of literature search strategy process.

A total of 2626 potential articles were screened at the first screening, and 19 observational studies with 6256 participants were finally included. A flow diagram depicting the process of literature search strategy is shown in Figure 1. Among the 19 studies enrolled, 7 studies investigated the clinical outcomes of DES-ISR versus BMS-ISR after the treatment of DEB, and the remaining 12 studies compared the clinical outcomes of DES-ISR with BMS-ISR after the treatment of DES. Among the participants enrolled, 2514 patients were with DES-ISR, and 3742 patients were with BMS-ISR.  Table 1 describes the main characteristics of the included studies. The mean follow-up time ranged from 8 to 72 months. Baseline characteristics of the enrolled patients are shown in Table 2. Risk of bias assessment is listed in Table 3.

**Figure 2:**
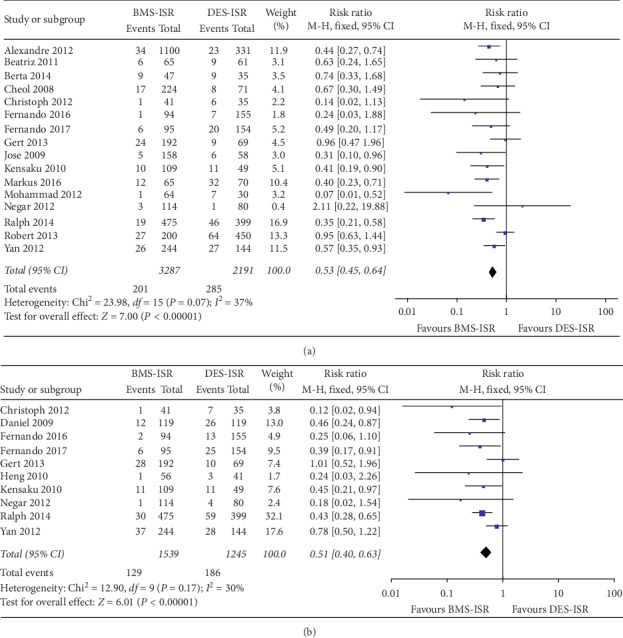
Forest plot with RR for BMS-ISR versus DES-ISR: (a) TLR, (b) TVR.

**Figure 3:**
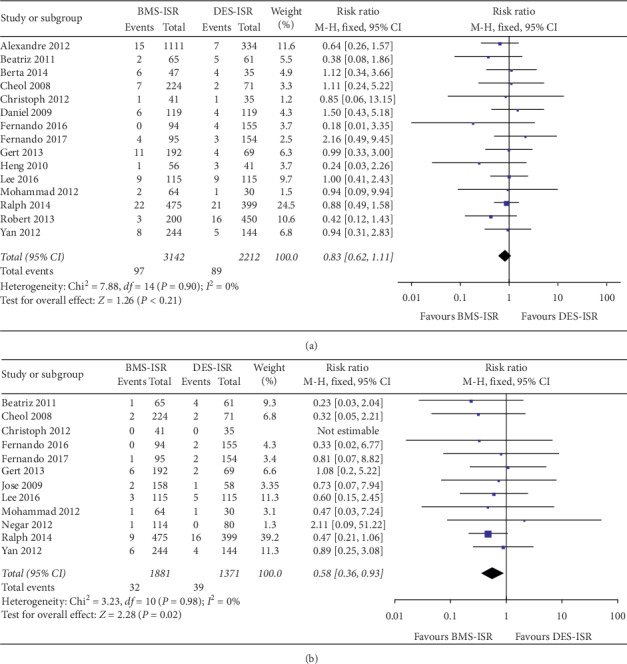
Forest plot with RR for BMS-ISR versus DES-ISR: (a) ACD, (b) CD.

**Figure 4:**
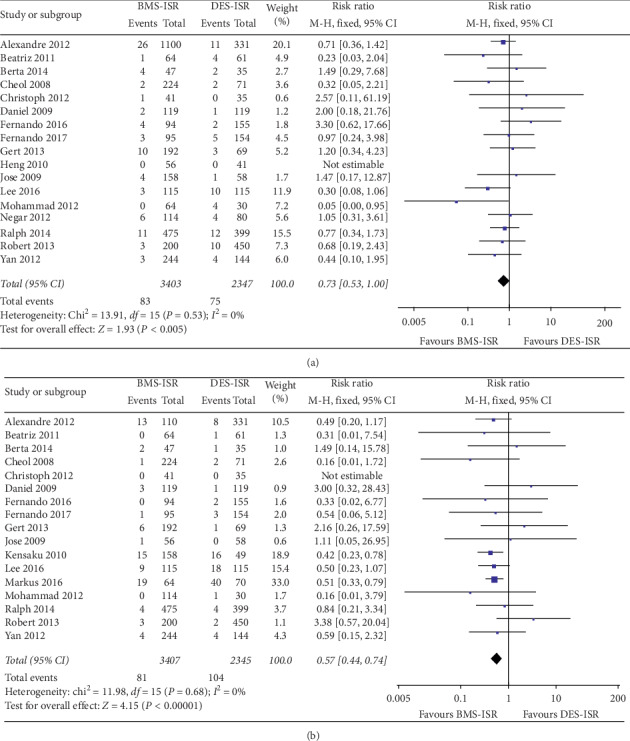
Forest plot with RR for BMS-ISR versus DES-ISR: (a) MI, (b) ST/RS-ISR.

**Figure 5:**
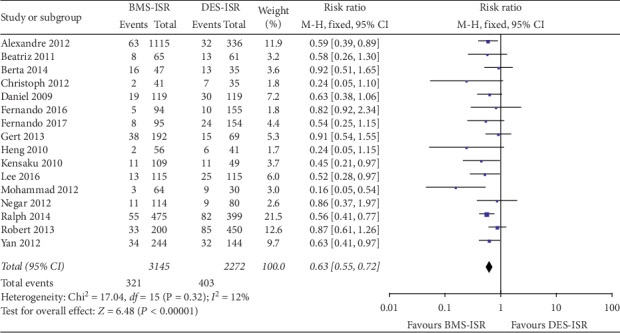
Forest plot with RR for BMS-ISR versus DES-ISR : MACEs.

In terms of the clinical outcomes, 16 studies including 5478 patients contributed to analysis of the overall rate of TLR, which was significantly higher in the DES-ISR group than in the BMS-ISR group (RR: 0.53, 95% CI: 0.45–0.64, *P* < 0.00001, Figure 2(a)); 10 studies with 2784 patients contributed to analysis of the overall rate of TVR, which was significantly higher in the DES-ISR group than in the BMS-ISR group (RR: 0.51, 95% CI: 0.40–0.63, *P* < 0.00001, Figure 2(b)); 15 studies including 5354 patients contributed to analysis of the overall rate of ACD, which was similar between the two groups (RR: 0.83, 95% CI: 0.62–1.11, *P*=0.21, Figure 3(a)); 12 studies with 3252 patients contributed to analysis of the overall rate of CD, which was significantly higher in the DES-ISR group than in the BMS-ISR group (RR: 0.58, 95% CI: 0.36–0.93, *P*=0.02, Figure 3(b)); 17 studies with 5750 patients reported the rates of MI, and results showed that patients with DES-ISR had higher rates of MI than patients with BMS-ISR, although not statistically significant (RR: 0.73, 95% CI: 0.53–1.00, *P*=0.05, Figure 4(a)); 17 studies reported the incidences of ST or RE-ISR, and the results showed that the rates were significantly higher in the DES-ISR group than in the BMS-ISR group (RR: 0.57, 95% CI: 0.44–0.74, *P* < 0.0001, Figure 4(b)); 16 studies with 5417 patients contributed to the analysis of the overall rate of MACEs, which was markedly higher in the DES-ISR group compared with the BMS-ISR group (RR: 0.63, 95% CI: 0.55–0.72, *P* < 0.00001, Figure 5).

Subgroup analyses showed that the incidences of TLR (*P* < 0.00001, Supplemental Figure 1A), TVR (*P*=0.0003, Supplemental Figure 1B), ST/RE-ISR (*P*=0.01, Supplemental Figure 2C), and MACEs (*P* < 0.00001, Supplemental Figure 3) at the long-term follow-up were markedly higher in the DES-ISR group than in the BMS-ISR group after treated by DES, but the rates of ACD (*P*=0.15, Supplemental Figure 1C), CD (*P*=0.42, Supplemental Figure 2A) and MI (*P*=0.21, Supplemental Figure 2B) were similar between the two groups.

Similarly, patients with DES-ISR had higher rates of TLR (*P* < 0.00001, Supplemental Figure 4A), TVR (*P* < 0.00001, Supplemental Figure 4B), CD (*P*=0.02, Supplemental Figure 5A), ST/RE-ISR (*P*=0.0007, Supplemental Figure 5C), and MACEs (*P* < 0.00001, Supplemental Figure 6) at the long-term follow-up than patients with BMS-ISR after treated by DEB, but no significant differences were found between the two groups in the rates of ACD (*P*=0.74, Supplemental Figure 4C) and MI (*P*=0.013, Supplemental Figure 5B).

## 4. Discussion

This is the first meta-analysis to investigate the long-term clinical outcomes after treatment for DES-ISR compared with BMS-ISR. What we found was that patients with DES-ISR had poorer clinical outcomes than patients with BMS-ISR after treated by DES or DEB.

The reasons of these findings are not fully understood, and possible explanations are as follows: first, different pathological features of the two types of ISR lesion may result in different outcomes. The homogeneous type mainly composed of the smooth muscle cells with collagen fibers is predominant in the BMS-ISR lesions, while layered type that comprises proteoglycans, inflammatory cells, and fibrinoids is the main pattern of the DES-ISR lesions. Besides, neoatherosclerosis occurs more frequently and earlier in DES-ISR lesions than in BMS-ISR lesions [27, 28]. Nagoshi et al. [26] evaluated the efficiency of BA for homogeneous and layered lesions, and the results showed that after BA, reduction in neointimal tissue area was significantly smaller in homogeneous lesions than in layered lesions, suggesting that layered ISR tissue may respond better to BA than those homogeneous ISR tissue. Based on this concept, one speculated that different patterns of neointimal tissue may also have different responses to DES or DEB (DES or DEB is more effective in homogeneous type but might be less effective in layered type tissue and more effective in classical neointimal proliferation but less effective in neoatherosclerosis). Further studies with optical coherence tomography (OCT) or intravascular ultrasound (IVUS) are required to confirm these speculations.

Another possible explanation is that the vascular wall of DES-ISR may have poorer response to the repeated anti-inflammatory and antiproliferative drugs which were covered by DES or DEB after the wall shows resistance to the beneficial effects of DES in a de novo lesion by developing ISR. However, the lesions of BMS-ISR are “drug-naive,” which may create a potential milieu for the anti-inflammatory and proliferative drugs to play their roles richly after DES or DEB implanted to the lesions [8,9,14].

Finally, the selection bias of patients may also lead to the difference of outcomes between DES-ISR and BMS-ISR. As we known, the use of DES has significantly reduced the incidence of ISR compared to BMS, but with the growing application of DES in the complex circumstances, DES-ISR has also increased [1, 3]. Therefore, the majority of DES-ISR patients in our enrolled studies are usually those who have more adverse characteristics such as diabetes, ACS, and more complex or severe lesions than BMS-ISR patients, which may impair the efficiency of DES or DEB for patient with DES-ISR [9, 24].

The main findings of our study suggest that we should pay more attention to how to prevent patients with DES-ISR from undergoing unfavorable outcomes after treated by DES or DEB. In other words, concentrating on the predictors of outcomes of DES-ISR after treated by DES or DEB is required. Abizaid et al. [24] found that the independent predictors of TLR after using SES for the treatment of DES-ISR were diabetes mellitus in advanced stage (*P*=0.001), postprocedure diameter stenosis <20% (*P* < 0.001), bifurcation lesion treated with no less than 2 stents (*P*=0.004), and the total number of lesions treated (*P*=0.009). Besides, independent predictors of MACEs after using SES for the treatment of DES-ISR were diabetes mellitus in advanced stage (*P* < 0.001), postprocedure residual stenosis (*P*=0.001), and bifurcation lesion treated with 2 stents (*P* < 0.015). In another study, the associations of TLR following the use of DEB for the treatment of patients with DES-ISR included end stage renal disease on maintenance hemodialysis (*P*=0.047) and previous DEB failure (*P* < 0.001) [7]. Moreover, it is also indispensable to prevent DES-ISR from occurring. There are different kinds of risk factors associated with DES-ISR, including female gender, diabetes mellitus, renal failure, and complex lesions such as type C lesions, calcified lesion, long lesion, and small diameter vessel. For patients with high risk of DES-ISR, OCT, IVUS, and fractional flow reserve (FFR) may be helpful for clinicians to decide whether a DES implantation or not and to avoid the procedure-related factors such as stent fracture and stent underexpansion [3,29,30].

Although our study demonstrated that DES and DEB had less efficiency and safety for DES-ISR than for BMS-ISR, there are no other better choices than DES and DEB. A meta-analysis comparing the efficacy of DES, DEB, and BA for DES-ISR showed that both DES and DEB were superior to BA, but there were no significant differences between the DES group and the DEB group [31]. Another meta-analysis found no differences in the rates of TLR, CD, MI, ST, and MACEs between the DEB group and the DES group, but with meta-analysis of clinical trials only, the TLR rate was significantly reduced in the DES group (*P*=0.015) [32]. Recently, several studies were conduct to investigate the efficiency of DEB versus DES stratified by the generation for DES-ISR, but there was no enough evidence to confirm which is better. PEPCAD China ISR trial was designed to compare first-generation DES (FG-DES) versus DEB for DES-ISR showed that the clinical outcomes at 1-year follow-up were similar between the two groups [33]. A multicenter randomized study involving 309 patients demonstrated that the rates of TLR, TVR, and MACEs at 1 year and 3 years were significantly lower in patients treated with second-generation DES (SG-DES) than those treated with DEB [34,35]. However, in another multicenter randomized trial enrolling 172 patients, the rates of TLR, TVR, ACD, MI, and ST at 1-year follow-up were comparable between the SG-DES group and the DEB group [36]. Recently, a meta-analysis comparing DEB versus SG-DES for the management of ISR was conducted and the subgroup analysis of DES-ISR showed that second-generation DES was associated with lower risk of TLR (*P*=0.004), TVR (*P*=0.012), and MACEs (*P*=0.043) than DEB, but the sample size of the subgroup is so small that the statistical power to evaluate the effective size may be not enough to properly compare the efficacy and safety of DEB and SG-DES in DES-ISR patients [37]. Whether DES (including FG-DES and SG-DES) or DEB is more effective for DES-ISR remains unclear. Further large-scale randomized trials are required to found out the answers.

Recently, several studies were conducted to investigate whether there were outcomes differences when DES-ISR treated by different types of DES. In the ISAR-DESIRE-2 study, 450 patients with sirolimus DES-ISR were randomly divided into resirolimus DES treatment group and paclitaxel DES treatment group, and the results showed that the rates of TLR (*P*=0.52), ACD (*P*=0.6), MI (*P*=0.53), and ST (*P*=0.67) at 1-year follow-up were similar between the two groups [38]. Similarly, in the RIBS III study, there were also no marked differences of the clinical outcomes between the hetero-DES and homo-DES group [39]. Whether using a different DES or a similar DES when DES-ISR occurs remains controversial. Besides, there were limited studies conducted to investigated differences between FG-DES and SG-DES for DES-ISR. The study of Song et al. [40] which included patients with diffuse type DES-ISR demonstrated that implantation of SES or EES had comparable efficiency and safety for the treatment of DES-ISR in terms of clinical outcomes at 1-year follow-up.

There are a number of alternative DEB devices that are available for DES-ISR. Colleran et al. [41] compared two different kinds of paclitaxel-coated balloons for DES-ISR; the results demonstrated that the clinical outcomes including TLR (*P*=0.91), ACD (*P*=0.73), MI (*P*=0.73), ST (*P*=0.34), and MACEs (*P*=0.91) at 1 year were similar between the BTHC-based PEB group and iopromide-based PEB group. In a multicenter randomized trial enrolling 50 patients with DES-ISR, the incidence of TLR, ACD, ST, and MACES up to 12 months did not differ between the sirolimus-coated balloon group and paclitaxel-coated balloon group [42].

Overall, with regard to the treatment for DES-ISR, whether DES or DEB, which kind of DES and DEB is more appropriated for DES-ISR remains unclear. Large-scale randomized trials are needed to determine the optimal strategies for DES-ISR.

### 4.1. Limitations

Firstly, the studies pooled in this analysis were all observational studies, which may decrease the validity of the study to a certain extent. Besides, there was a level of heterogeneity between the included studies due to different initial DES types. Finally, we did not analyze the angiographic outcomes because the pattern of quantitative coronary assessment was inconsistent, some were by in-segment pattern, and others were by in-stent pattern.

### 4.2. Conclusions

Our study demonstrated that patients with DES-ISR had worse clinical outcomes at the long-term follow-up than patients with BMS-ISR after the treatment of DES or DEB, suggesting that DES and DEB may be more effective for BMS-ISR than that for DES-ISR. Positive prevention of DES-ISR is indispensable and further studies concentrating on detecting the predictors of outcomes of DES-ISR are required.

## Figures and Tables

**Table 1:** Main characteristics of the included studies.

First author	Published year	Study type	TPN	Treatment	FU time	Endpoints
Berta	2014	Observational	82	DEB	28 months	TLR, ST, MI, MACE, death
Lee	2016	Observational	230	DEB	12 months	RE-ISR, MI, MACE, death, CD
Alfonso	2017	Observational	249	DEB	12 months	TLR, TVR, ST, MI, MACE, death, CD
Beatriz	2011	Observational	126	DEB	12 months	TLR, ST, MI, MACE, death, CD
Markus	2016	Observational	135	DEB	12 months	RE-ISR, TLR
Christoph	2012	Observational	81	DEB	12 months	TLR, TVR, ST, MI, MACE, death, CD
Ralph	2014	Observational	918	DEB	13 months	TLR, TVR, ST, MI, MACE, death, CD
Daniel	2009	Observational	238	DES	12 months	TVR, ST, MI, MACE, death
Robert	2013	Observational	650	DES	12 months	TLR, ST, MI, MACE, death
Negar	2012	Observational	194	DES	12 months	TLR, TVR, MI, MACE, CD
Jose	2009	Observational	216	DES	72 months	TLR, ST, MI, CD
Heng	2010	Observational	97	DES	28 months	TVR, MI, MACE, death
Fernando	2016	Observational	249	DES	12 months	TLR, TVR, ST, MI, MACE, death, CD
Mohammad	2012	Observational	94	DES	12 months	TLR, ST, MI, MACE, death, CD
Cheol	2008	Observational	295	DES	32 months	TLR, ST, MI, death, CD
Yan	2013	Observational	388	DES	42 months	TLR, TVR, ST, MI, MACE, death, CD
Kensaku	2010	Observational	158	DES	8 months	TLR, TVR, RE-ISR, MACE
Alexandre	2012	Observational	1590	DES	12 months	TLR, ST, MI, MACE, death
Gert	2013	Observational	266	DES	24 months	TLR, TVR, ST, MI, MACE, death, CD

TPN: total patient number; FU: follow-up; DEB: drug-eluting balloon; DES: drug-eluting stent; TLR: target lesion revascularization; TVR: target vessel revascularization; CD: cardiac death; MI: myocardial infarction; ST: stent thrombosis; RE-ISR: re-in-stent restenosis; MACE: major adverse cardiovascular event.

**Table 2:** Basic characteristics of the enrolled patients.

Study	Type of ISR	PN	Age (years)	Male (%)	HTN (%)	DM (%)	HLP (%)	Smoke (%)	ACS (%)
Berta	BMS	47	63.6 ± 10.2	51.1	97.9	38.3	87.2	23.4	25.6
	DES	35	62.7 ± 10.0	45.7	100.0	34.3	94.3	22.9	14.3
Lee	BMS	115	65.1 ± 10.4	77.4	75.7	50.4	67.0	43.5	82.6
	DES	115	63.5 ± 10.3	76.5	75.7	57.4	66.1	36.5	77.4
Fernando	BMS	95	67.0 ± 11.0	86.0	72.0	32.0	73.0	59.0	40.0
	DES	154	66.0 ± 10.0	82.0	71.0	49.0	71.0	58.0	52.0
Beatriz	BMS	65	66.2 ± 11.9	78.5	69.2	27.7	60.0	30.8	66.2
	DES	61	64.4 ± 10.2	88.5	80.3	39.3	77.0	29.5	34.4
Markus	BMS	65	59.9 ± 9.4	76.9	89.2	36.9	—	—	—
	DES	70	65.0 ± 8.7	71.4	87.1	41.4	—	—	—
Christoph	BMS	43	65.0 ± 8.8	79.1	81.4	25.6	81.4	—	14.0
	DES	38	67.0 ± 10.1	76.3	94.8	29.0	94.7	—	15.8
Ralph	BMS	499	66.9 ± 10.8	76.4	85.8	30.7	84.8	60.7	33.7
	DES	419	66.8 ± 10.5	72.1	84.7	38.2	86.2	57.8	31.3
Daniel	BMS	119	63.4 ± 10.9	68.9	90.8	40.5	93.2	16.8	63.9
	DES	119	64.4 ± 11.4	60.5	95.8	42.7	96.6	19.3	71.4
Robert	BMS	200	64.2 ± 10.6	78.5	54.0	29.0	56.0	11.0	—
	DES	450	66.7 ± 10.6	76.7	72.4	36.0	75.8	12.0	—
Negar	BMS	114	57.5 ± 9.9	67.5	48.2	25.4	75.4	21.9	51.9
	DES	80	56.4 ± 11.0	66.3	42.5	26.3	70.0	23.8	58.7
Jose	BMS	158	62.6 ± 11.5	72.8	78.5	32.9	67.1	10.8	24.1
	DES	58	59.5 ± 9.8	71.7	75.8	36.1	79.3	24.1	43.1
Heng	BMS	56	63.7 ± 11.9	80.4	64.3	26.8	—	—	48.2
	DES	41	65.7 ± 9.3	70.7	78.0	43.9	—	—	41.4
Fernando	BMS	94	64.0 ± 12.0	87.0	72.0	20.0	66.0	—	45.0
	DES	155	66.0 ± 10.0	84.0	78.0	42.0	78.0	—	51.0
Mohammad	BMS	64	67.9 ± 10.6	87.5	76.5	28.1	31.2	53.1	—
	DES	30	66.8 ± 11.9	73.3	86.0	43.3	16.6	50.0	—
Cheol	BMS	224	59.9 ± 10.6	76.3	50.0	31.9	—	22.3	41.5
	DES	71	58.7 ± 10.9	66.2	47.9	22.5	—	19.7	38.0
Yan	BMS	244	58.0 ± 10.9	85.2	66.0	27.5	52.9	53.7	59.9
	DES	144	57.4 ± 9.0	81.9	68.1	25.0	54.9	44.4	59.7
Kensaku	BMS	109	66.6 ± 10.8	84.0	73.0	33.0	41.0	17.0	—
	DES	49	67.0 ± 8.3	84.0	73.0	57.0	57.0	12.0	—
Alexandre	BMS	1235	63.2 ± 10.8	73.3	76.9	26.9	81.1	54.1	43.5
	DES	355	63.7 ± 10.5	70.4	71.3	39.4	79.8	54.1	44.2
Gert	BMS	196	65.5 ± 10.4	75.0	—	29.1	—	—	45.4
		7	65.6 ± 10.6	82.9	—	32.9	—	—	45.7

BMS: bare-metal stent; DES: drug-eluting stent; PN: patient number; HTN: hypertension; DM: diabetes mellitus; HLP: hyperlipemia; ACS: acute coronary syndrome; ISR: in-stent restenosis; -: not available.

**Table 3:** Risk of bias assessment.

Study	1	2	3	4	5	6	7	8	Total
Berta	A	A	A	A	A	B	A	A	8
Lee	A	A	A	B	A	B	A	A	7
Fernando	A	A	A	B	A	B	A	A	7
Beatriz	A	A	A	A	A	B	A	A	8
Markus	A	A	A	B	A	B	A	A	7
Christoph	A	A	A	A	A	B	A	B	8
Ralph	A	A	A	A	A	B	A	B	8
Daniel	A	A	A	B	A	B	A	A	7
Robert	A	A	A	B	A	B	A	A	7
Negar	A	A	A	B	A	B	A	A	7
Jose	A	A	A	B	A	B	A	A	7
Heng	A	A	A	B	A	B	A	A	7
Fernando	A	A	A	B	A	B	A	A	7
Mohammad	A	A	A	B	A	B	A	A	7
Cheol	A	A	A	A	A	B	A	A	8
Yan	A	A	A	B	A	B	A	A	7
Kensaku	A	A	A	A	A	B	A	A	8
Alexandre	A	A	A	B	A	B	A	C	6
Gert	A	A	A	B	A	B	A	B	7

1: representativeness of the exposed cohort; 2: selection of the nonexposed cohort; 3: ascertainment of exposure; 4: outcome of interest was not present at the beginning of study; 5: comparability of cohorts; 6: assessment of outcome; 7: long enough follow-up; 8: adequacy of follow-up; A: 1 score; B: 0/1 score; C: 0 score.

## Data Availability

All data used to support the findings of our study are included within the article.

## Supplementary Materials

Supplementary Materials Supplement Figure 1: forest plot with RR for BMS-ISR versus DES-ISR after treated by DES: (A) TLR, (B) TVR, and (C) ACD; Supplement Figure 2: forest plot with RR for BMS-ISR versus DES-ISR after treated by DES: (A) CD, (B) MI, and (C) ST/RE-ISR; Supplement Figure 3: forest plot with RR for BMS-ISR versus DES-ISR after treated by DES : MACES; Supplement Figure 4: forest plot with RR for BMS-ISR versus DES-ISR after treated by DEB: (A) TLR, (B) TVR, and (C) ACD; Supplement Figure 5: forest plot with RR for BMS-ISR versus DES-ISR after treated by DEB: (A) CD, (B) MI, and (C) ST/RE-ISR; Supplement Figure 6: forest plot with RR for BMS-ISR versus DES-ISR after treated by DEB : MACES.
