# Supplementary material for: Clinical Outcomes of Drug-Eluting versus Bare-Metal In-Stent Restenosis after the Treatment of Drug-Eluting Stent or Drug-Eluting Balloon: A Systematic Review and Meta-Analysis
Source: J Interv Cardiol. 2020 Jun 26;2020:8179849. doi: 10.1155/2020/8179849 (PMC7336236; doi:10.1155/2020/8179849)
Supplement: Supplementary Materials — Supplement Figure 1: forest plot with RR for BMS-ISR versus DES-ISR after treated by DES: (A) TLR, (B) TVR, and (C) ACD; Supplement Figure 2: forest plot with RR for BMS-ISR versus DES-ISR after treated by DES: (A) CD, (B) MI, and (C) ST/RE-ISR; Supplement Figure 3: forest plot with RR for BMS-ISR versus DES-ISR after treated by DES : MACES; Supplement Figure 4: forest plot with RR for BMS-ISR versus DES-ISR after treated by DEB: (A) TLR, (B) TVR, and (C) ACD; Supplement Figure 5: forest plot with RR for BMS-ISR versus DES-ISR after treated by DEB: (A) CD, (B) MI, and (C) ST/RE-ISR; Supplement Figure 6: forest plot with RR for BMS-ISR versus DES-ISR after treated by DEB : MACES. [file 8179849.f1.pdf]

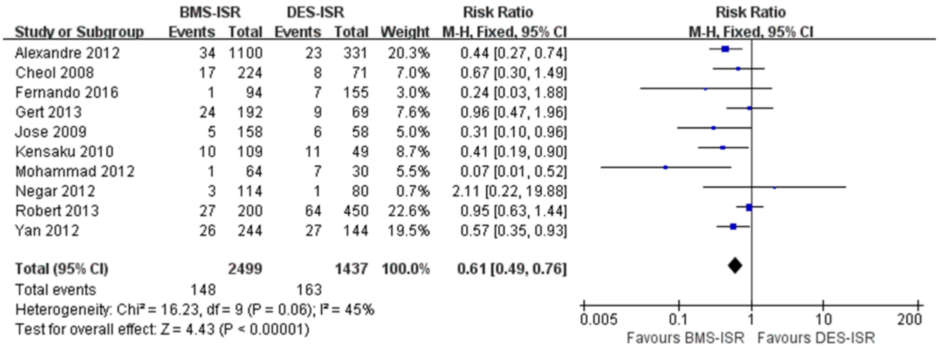

(A)

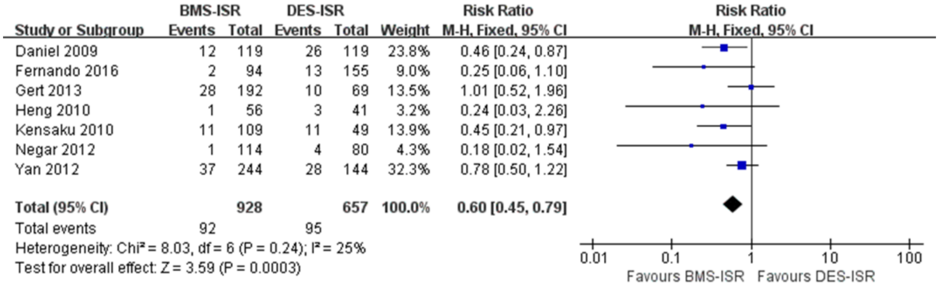

(B)

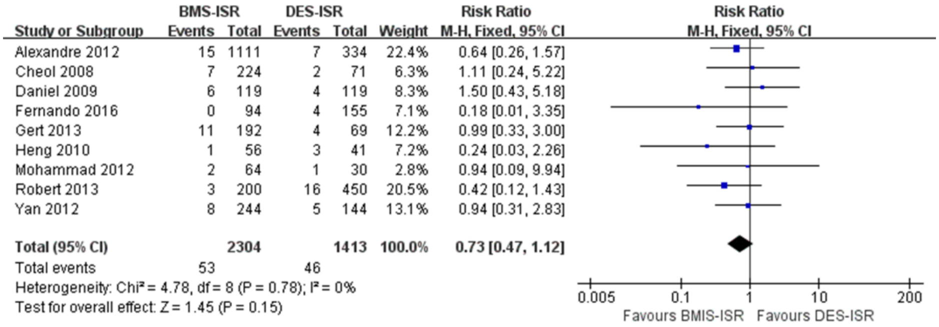

(C)

Supplement figure 1: Forest plot with RR for BMS-ISR vs DES-ISR after treated by DES: (A) TLR (B) TVR (C) ACD

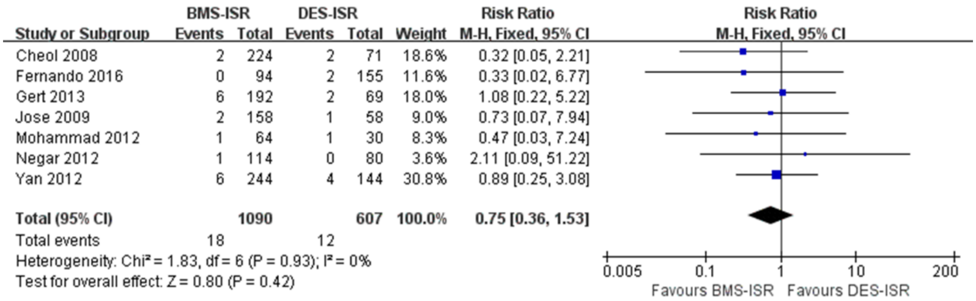

(A)

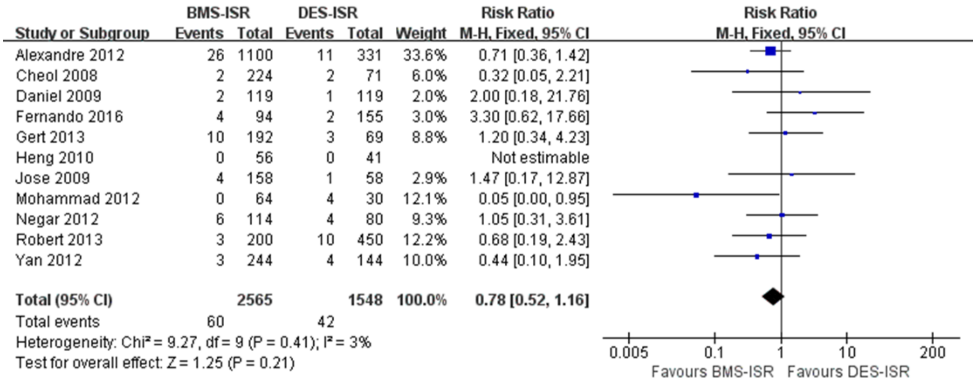

(B)

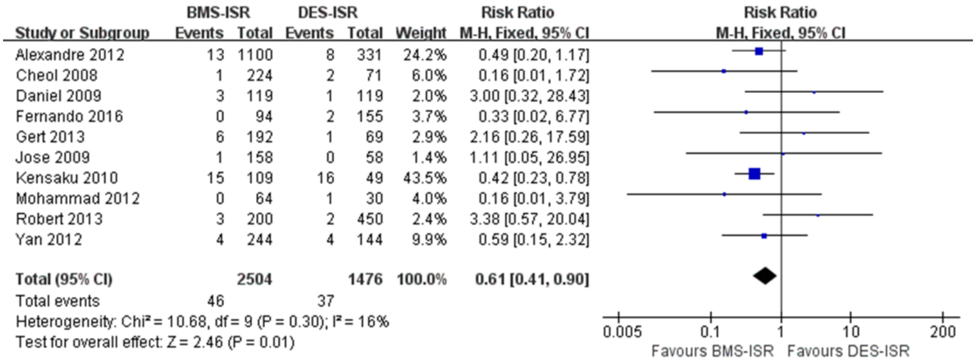

(C)

Supplement figure 2: Forest plot with RR for BMS-ISR vs DES-ISR after treated by DES: (A) CD (B) MI (C) ST/RE-ISR

Study or Subgroup	BMS-ISR Events	BMS-ISR Total	DES-ISR Events	DES-ISR Total	Weight	Risk Ratio M-H, Fixed, 95% CI
Alexandre 2012	63	1115	32	336	20.0%	0.59 [0.39, 0.89]
Daniel 2009	19	119	30	119	12.2%	0.63 [0.38, 1.06]
Fernando 2016	5	94	10	155	3.1%	0.82 [0.29, 2.34]
Gert 2013	38	192	15	69	9.0%	0.91 [0.54, 1.55]
Heng 2010	2	56	6	41	2.8%	0.24 [0.05, 1.15]
Kensaku 2010	11	109	11	49	6.2%	0.45 [0.21, 0.97]
Mohammad 2012	3	64	9	30	5.0%	0.16 [0.05, 0.54]
Negar 2012	11	114	9	80	4.3%	0.86 [0.37, 1.97]
Robert 2013	33	200	85	450	21.2%	0.87 [0.61, 1.26]
Yan 2012	34	244	32	144	16.3%	0.63 [0.41, 0.97]
**Total (95% CI)**		**2307**		**1473**	**100.0%**	**0.67 [0.56, 0.80]**
Total events	219		239			

Heterogeneity: $Chi^2 = 12.31$, $df = 9$ ($P = 0.20$); $I^2 = 27\%$

Test for overall effect: $Z = 4.44$ ($P < 0.00001$)

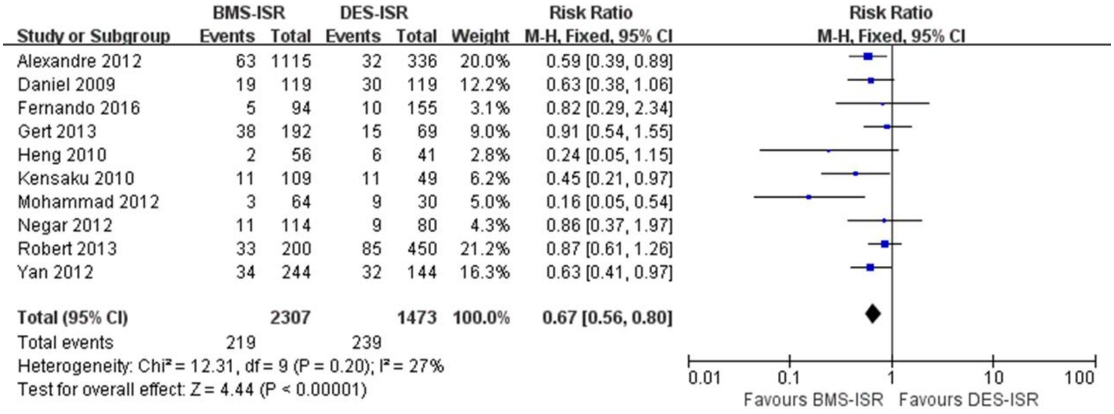

Supplement figure 3: Forest plot with RR for BMS-ISR vs DES-ISR after treated by DES: MACES

Study or Subgroup	BMS-ISR Events	BMS-ISR Total	DES-ISR Events	DES-ISR Total	Weight	Risk Ratio M-H, Fixed, 95% CI
Beatriz 2011	6	65	9	61	7.6%	0.63 [0.24, 1.65]
Berta 2014	9	47	9	35	8.4%	0.74 [0.33, 1.68]
Christoph 2012	1	41	6	35	5.3%	0.14 [0.02, 1.13]
Fernando 2017	6	95	20	154	12.5%	0.49 [0.20, 1.17]
Markus 2016	12	65	32	70	25.2%	0.40 [0.23, 0.71]
Ralph 2014	19	475	46	399	40.9%	0.35 [0.21, 0.58]
**Total (95% CI)**		**788**		**754**	**100.0%**	**0.42 [0.31, 0.57]**
Total events	53		122			

Heterogeneity: Chi² = 4.23, df = 5 (P = 0.52); I² = 0%
Test for overall effect: Z = 5.60 (P < 0.00001)

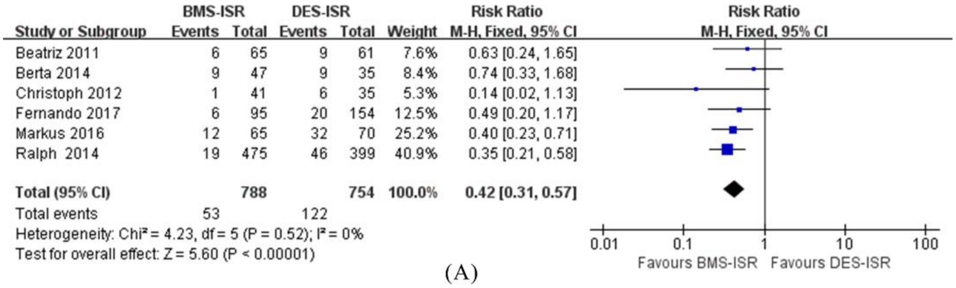

(A)

Study or Subgroup	BMS-ISR Events	BMS-ISR Total	DES-ISR Events	DES-ISR Total	Weight	Risk Ratio M-H, Fixed, 95% CI
Christoph 2012	1	41	7	35	8.3%	0.12 [0.02, 0.94]
Fernando 2017	6	95	25	154	21.0%	0.39 [0.17, 0.91]
Ralph 2014	30	475	59	399	70.7%	0.43 [0.28, 0.65]
**Total (95% CI)**		**611**		**588**	**100.0%**	**0.39 [0.27, 0.57]**
Total events	37		91			

Heterogeneity: Chi² = 1.41, df = 2 (P = 0.50); I² = 0%
Test for overall effect: Z = 4.96 (P < 0.00001)

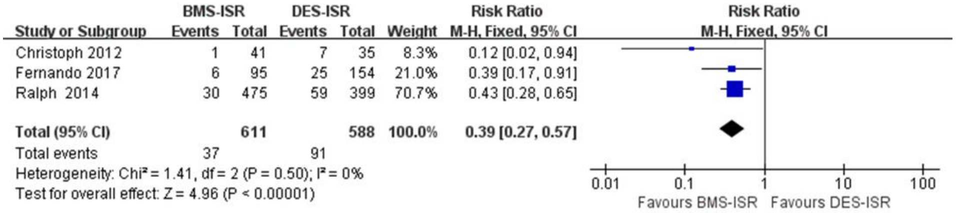

(B)

Study or Subgroup	BMS-ISR Events	BMS-ISR Total	DES-ISR Events	DES-ISR Total	Weight	Risk Ratio M-H, Fixed, 95% CI
Beatriz 2011	2	65	5	61	11.5%	0.38 [0.08, 1.86]
Berta 2014	6	47	4	35	10.2%	1.12 [0.34, 3.66]
Christoph 2012	1	41	1	35	2.4%	0.85 [0.06, 13.15]
Fernando 2017	4	95	3	154	5.1%	2.16 [0.49, 9.45]
Lee 2016	9	115	9	115	20.0%	1.00 [0.41, 2.43]
Ralph 2014	22	475	21	399	50.8%	0.88 [0.49, 1.58]
**Total (95% CI)**		**838**		**799**	**100.0%**	**0.93 [0.62, 1.40]**
Total events	44		43			

Heterogeneity: Chi² = 2.64, df = 5 (P = 0.76); I² = 0%
Test for overall effect: Z = 0.33 (P = 0.74)

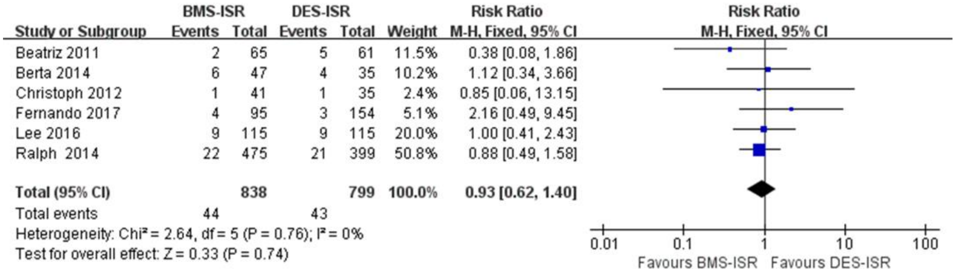

(C)

Supplement figure 4: Forest plot with RR for BMS-ISR vs DES-ISR after treated by DEB: (A) TLR (B) TVR (C) ACD

Study or Subgroup	BMS-ISR Events	BMS-ISR Total	DES-ISR Events	DES-ISR Total	Weight	Risk Ratio M-H, Fixed, 95% CI
Beatriz 2011	1	65	4	61	14.7%	0.23 [0.03, 2.04]
Christoph 2012	0	41	0	35		Not estimable
Fernando 2017	1	95	2	154	5.4%	0.81 [0.07, 8.82]
Lee 2016	3	115	5	115	17.8%	0.60 [0.15, 2.45]
Ralph 2014	9	475	16	399	62.0%	0.47 [0.21, 1.06]
**Total (95% CI)**		**791**		**764**	**100.0%**	**0.48 [0.25, 0.90]**
Total events	14		27			

Heterogeneity: Chi² = 0.70, df = 3 (P = 0.87); I² = 0%
Test for overall effect: Z = 2.27 (P = 0.02)

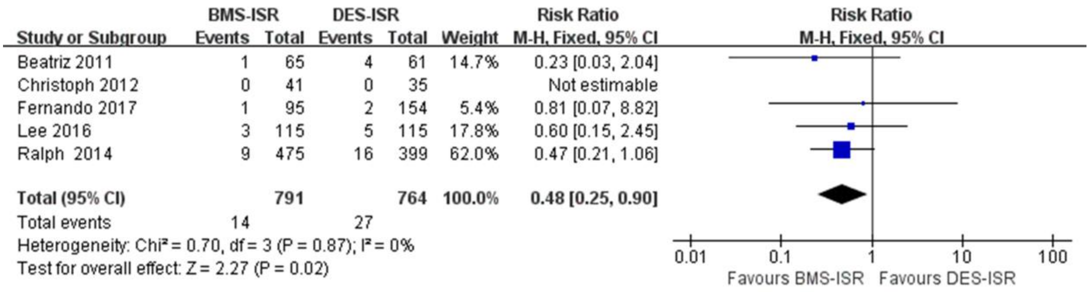

(A)

Study or Subgroup	BMS-ISR Events	BMS-ISR Total	DES-ISR Events	DES-ISR Total	Weight	Risk Ratio M-H, Fixed, 95% CI
Beatriz 2011	1	65	4	61	12.2%	0.23 [0.03, 2.04]
Berta 2014	4	47	2	35	6.8%	1.49 [0.29, 7.68]
Christoph 2012	1	41	0	35	1.6%	2.57 [0.11, 61.19]
Fernando 2017	3	95	5	154	11.3%	0.97 [0.24, 3.98]
Lee 2016	3	115	10	115	29.6%	0.30 [0.08, 1.06]
Ralph 2014	11	475	12	399	38.6%	0.77 [0.34, 1.73]
**Total (95% CI)**		**838**		**799**	**100.0%**	**0.67 [0.39, 1.12]**
Total events	23		33			

Heterogeneity: Chi² = 4.45, df = 5 (P = 0.49); I² = 0%
Test for overall effect: Z = 1.52 (P = 0.13)

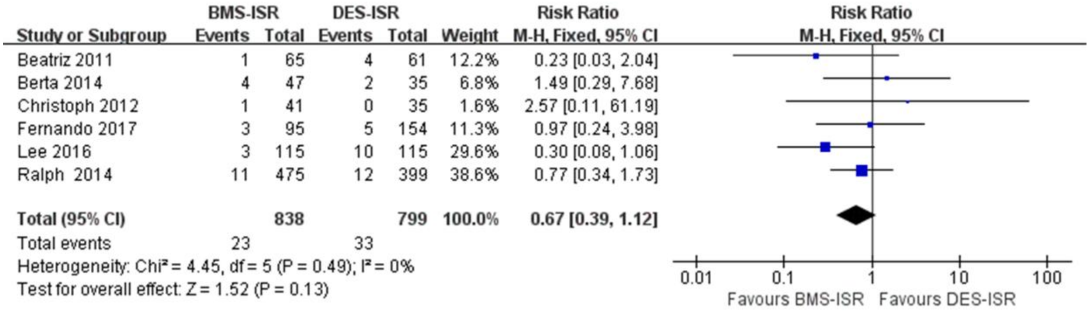

(B)

Study or Subgroup	BMS-ISR Events	BMS-ISR Total	DES-ISR Events	DES-ISR Total	Weight	Risk Ratio M-H, Fixed, 95% CI
Beatriz 2011	0	65	1	61	2.3%	0.31 [0.01, 7.54]
Berta 2014	2	47	1	35	1.7%	1.49 [0.14, 15.78]
Christoph 2012	0	41	0	35		Not estimable
Fernando 2017	1	95	3	154	3.5%	0.54 [0.06, 5.12]
Lee 2016	9	115	18	115	27.3%	0.50 [0.23, 1.07]
Markus 2016	19	65	40	70	58.5%	0.51 [0.33, 0.79]
Ralph 2014	4	475	4	399	6.6%	0.84 [0.21, 3.34]
**Total (95% CI)**		**903**		**869**	**100.0%**	**0.54 [0.38, 0.77]**
Total events	35		67			

Heterogeneity: Chi² = 1.32, df = 5 (P = 0.93); I² = 0%
Test for overall effect: Z = 3.37 (P = 0.0007)

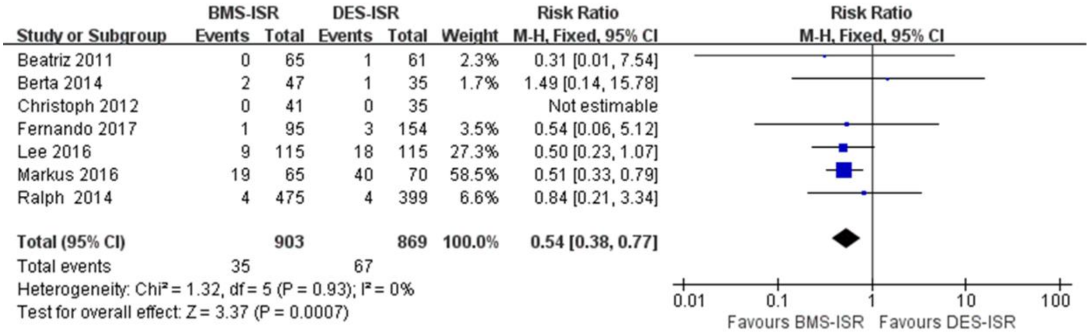

(C)

Supplement figure 5: Forest plot with RR for BMS-ISR vs DES-ISR after treated by DEB: (A) CD (B) MI (C) ST/RE-ISR

	BMS-ISR		DES-ISR			Risk Ratio
Study or Subgroup	Events	Total	Events	Total	Weight	M-H, Fixed, 95% CI
Beatriz 2011	8	65	13	61	8.0%	0.58 [0.26, 1.30]
Berta 2014	16	47	13	35	8.9%	0.92 [0.51, 1.65]
Christoph 2012	2	41	7	35	4.5%	0.24 [0.05, 1.10]
Fernando 2017	8	95	24	154	10.9%	0.54 [0.25, 1.15]
Lee 2016	13	115	25	115	14.9%	0.52 [0.28, 0.97]
Ralph 2014	55	475	82	399	53.0%	0.56 [0.41, 0.77]
**Total (95% CI)**		**838**		**799**	**100.0%**	**0.57 [0.46, 0.72]**
Total events	102		164			

Heterogeneity: $Chi^2 = 3.83$, df = 5 (P = 0.57); $I^2 = 0\%$
Test for overall effect: Z = 4.80 (P < 0.00001)

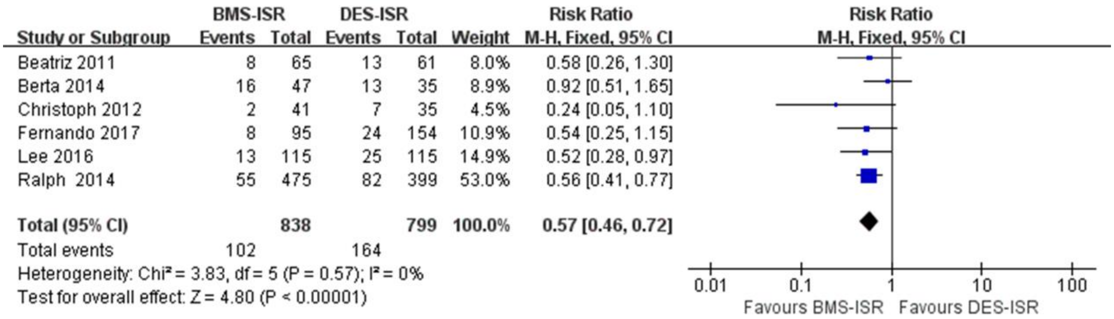

Supplement figure 6: Forest plot with RR for BMS-ISR vs DES-ISR after treated by DEB: MACES
